# The anticoagulation dilemma: A case report of pulmonary embolism in a gastric cancer patient with concurrent gastrointestinal bleeding and thrombocytopenia

**DOI:** 10.1097/MD.0000000000050395

**Published:** 2026-08-28

**Authors:** Xiaolin Yun, Tiantian Wang, Caixia Dong, Wenjun Yang, Xiaozhao Zhuang, Chenxin Zeng, Shuzhen Huang, Difei Yao

**Affiliations:** a Department of Pharmacy, Hainan General Hospital (Hainan Affiliated Hospital of Hainan Medical University), Haikou, Hainan, China; b Department of Pharmacy, The Second Affiliated Hospital of Zhejiang University School of Medicine, Hangzhou, Zhejiang, China; c Department of Medical Oncology, The Second Affiliated Hospital of Zhejiang University School of Medicine, Hangzhou, Zhejiang, China; d Research Center for Clinical Pharmacy, Zhejiang University, Hangzhou, Zhejiang, China; e Department of Radiology, Hainan General Hospital, Hainan Affiliated Hospital of Hainan Medical University, Haikou, Hainan, China; f Department of Pharmacy, Affiliated Jinhua Hospital, Zhejiang University School of Medicine, Jinhua, Zhejiang, China; g Department of Pharmacy, Wenzhou Central Hospital, Wenzhou, Zhejiang, China.

**Keywords:** anticoagulation, case report, gastrointestinal bleeding, pulmonary embolism, thrombocytopenia

## Abstract

**Rationale:**

Venous thromboembolism significantly contributes to the morbidity and mortality of patients with cancer, with fatal pulmonary embolism (PE) being 3 times more common in patients with cancer than in those without cancer. Delayed initiation of anticoagulant treatment in PE is correlated with increased mortality rates. Managing PE in this population is challenging because of the heightened risk of bleeding and thrombocytopenia, with limited guidance available in the current clinical guidelines.

**Patient concerns:**

A 60-year-old female weighing 86 kg was admitted to our hospital to initiate chemotherapy for advanced gastric cancer with gastric bleeding, accompanied by liver and abdominal lymph node metastases. Catheter-associated right femoral vein thrombosis and subsequent PE occurred during anticancer treatment, accompanied by a further decline in platelet count (PLT).

**Diagnoses:**

Catheter-associated right femoral vein thrombosis and subsequent PE occurred during anticancer treatment in a patient with gastric bleeding and liver/abdominal lymph node metastases, accompanied by a further decline in PLT.

**Interventions:**

Anticoagulation therapy was transitioned from enoxaparin to fondaparinux with careful dose titration based on the gastrointestinal bleeding activity, hemoglobin levels, and PLT.

**Outcomes:**

By dynamically adjusting anticoagulation therapy based on the patient’s evolving condition and bleeding status at different stages, we successfully treated the patient’s PE, ensuring control of the risk of gastrointestinal bleeding and restoration of the PLT.

**Lessons:**

This case underscores the importance of individualized anticoagulation strategies in patients with cancer and PE, particularly those at high risk of bleeding or heparin-induced thrombocytopenia. This highlights the need for further research and clinical guidelines to optimize the management of venous thromboembolism in this vulnerable population. However, owing to the lack of relevant laboratory testing resources at our medical institution, we were unable to confirm this through laboratory verification.

## 1. Introduction

Venous thromboembolism (VTE), particularly pulmonary embolism (PE), is a severe complication in patients with cancer and a leading cause of cancer-related mortality.^[1]^ Managing PE in this population is challenging due to the heightened risk of bleeding and thrombocytopenia, with limited guidance available in current clinical guidelines. We present a case of a patient with gastric cancer and PE who developed gastrointestinal bleeding and showed clinical features consistent with heparin-induced thrombocytopenia (HIT), significantly complicating her clinical management. Through a customized strategy that included switching to fondaparinux, adjusting its dosage, and implanting an inferior vena cava filter, the patient’s PE was successfully managed without aggravating the bleeding or thrombocytopenia.

## 2. Case presentation

**Figure 1.**
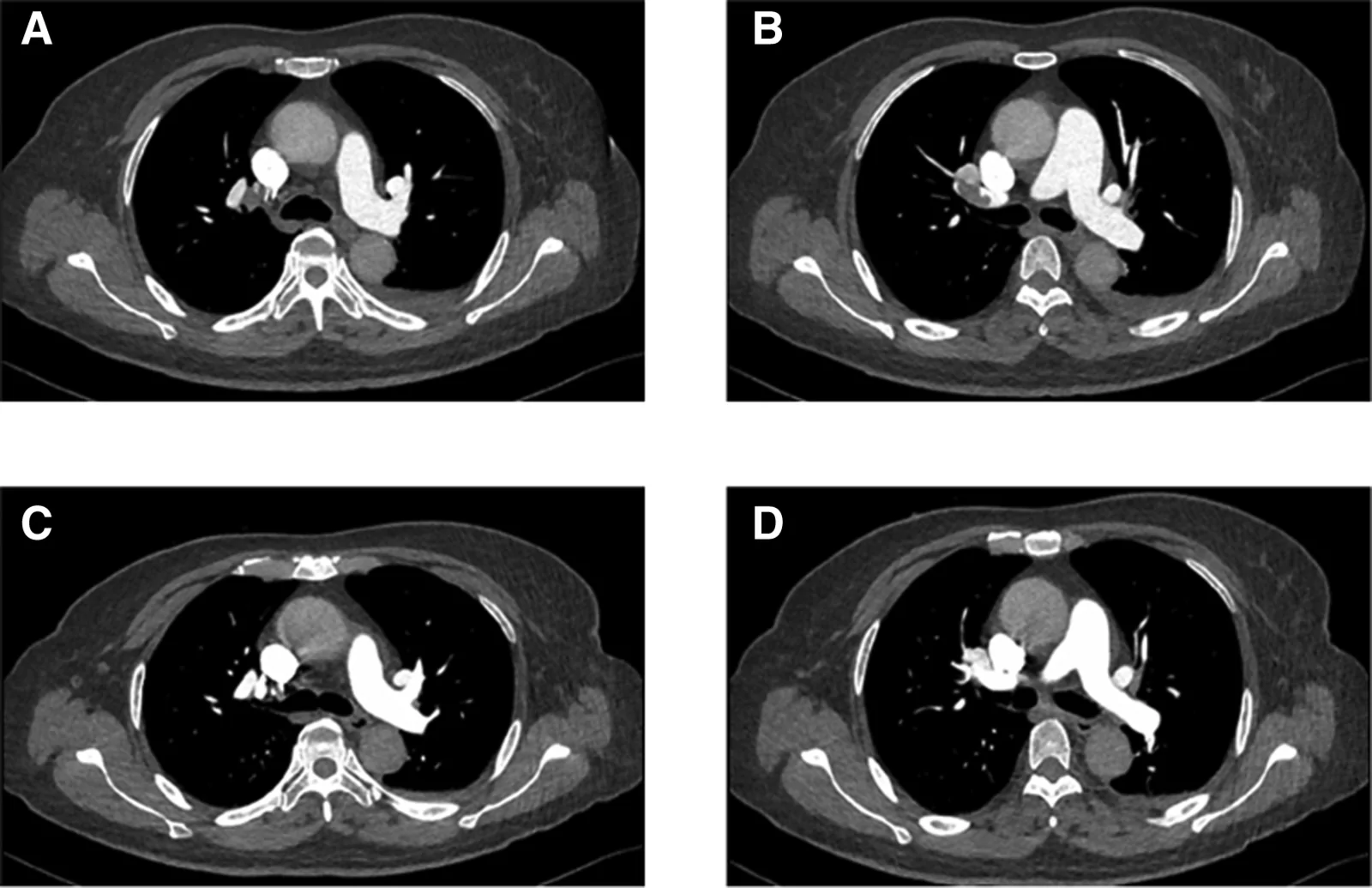
Right upper lobar pulmonary artery trunk and apical segment branch pulmonary artery embolism (A and B). The pulmonary embolism resolved (C and D).

**Figure 2.**
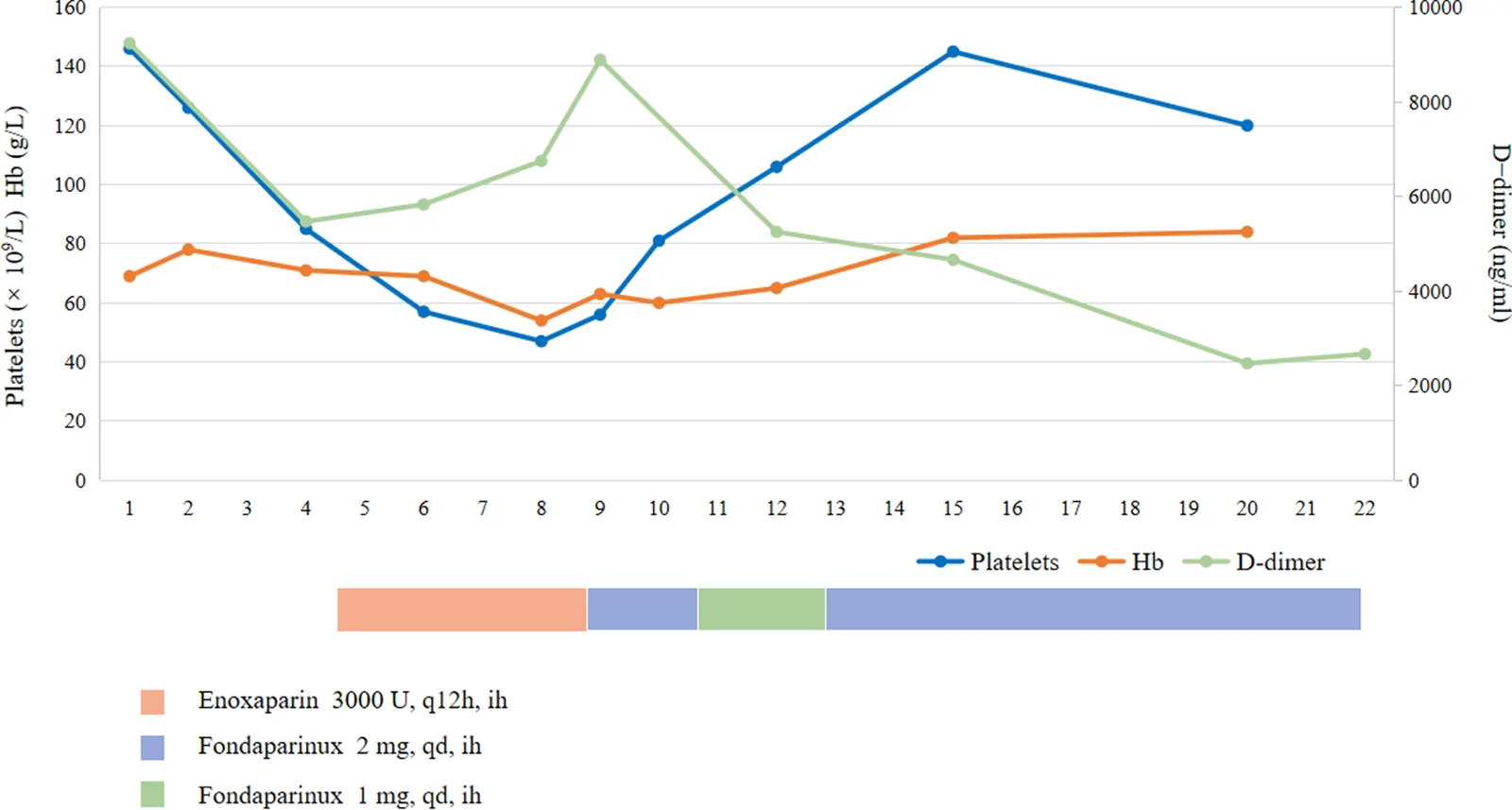
The clinical course of treatment.

A 60-year-old female weighing 86 kg was admitted to our hospital to initiate chemotherapy for advanced gastric cancer, which presented with gastric bleeding, accompanied by liver and abdominal lymph node metastases. Ultrasound imaging (June 19, 2023) revealed catheter-associated thrombosis in the right femoral vein. On the first day of admission, the patient’s platelet count (PLT) was 146 × 10^9^/L, hemoglobin (HB) was 69 g/L, and D-dimer was 9240 μg/L. The stool occult blood test was also positive. The patient received intravenous esomeprazole for gastric protection and was transfused with 2 units of packed red blood cells. Anticoagulation is not recommended in patients with a history of gastric cancer with bleeding and persistently low HB levels. On the second day, the patient received the first cycle of the FOLFOX regimen, combined with nivolumab. By the fifth day of admission, the PLT had decreased to 85 × 10^9^/L, with concurrent arrhythmia detected on electrocardiography and left atrial enlargement on echocardiography. Subsequent computed tomography pulmonary angiography (Fig. 1A and B) confirmed the diagnosis of PE. Following a pulmonology consultation, the recommended treatment plan included subcutaneous injections of 3000U of enoxaparin every 12 hours for anticoagulation, coupled with fasting to mitigate the risk of gastrointestinal bleeding. By day 8, the PLT had further dropped to a low of 47 × 10^9^/L. Considering that the patient had been on heparin for 5 days and the 4T score was 4, there was a moderate suspicion of HIT. We ceased enoxaparin, and the patient’s anticoagulation regimen was transitioned to 2 mg fondaparinux once daily. An inferior vena cava filter was surgically implanted in the patient. However, despite transfusion of packed red blood cells on the ninth day, the HB level did not significantly increase, and the patient continued to experience melena. Consequently, we reduced the fondaparinux dose to 1 mg once daily. On the 12th day, the patient started fasting, and a somatostatin pump was implanted to control gastrointestinal bleeding. On the 13th day, bloody stools ceased, and we adjusted the fondaparinux to 2 mg daily. On the 17th day, the patient received the second cycle of antitumor treatment. On the 22nd day, the patient began to take fluids. Subsequently, she was discharged with 2 mg fondaparinux once daily. Although lower limb venous thrombosis persisted, a follow-up computed tomography pulmonary angiography on July 24th revealed no PE (Fig. 1C and D). D-dimer levels decreased to 2600 μg/L, and CA125 showed a significant reduction compared with previous measurements. The clinical course of the treatment is shown in Figure 2.

## 3. Discussion

Cancer-associated thrombosis, a major cause of mortality in patients with cancer, most frequently manifests as VTE, which comprises deep vein thrombosis and PE. In China, a nationwide hospital-based study reported PE incidence and mortality rates of 14.19 and 1.00 per 100,000 population, respectively. Notably, 18.6% of these PE patients had coexisting malignancy; gastric cancer ranked among the top 3 cancer types and accounted for 1.3% of the total PE population.^[2]^ VTE contributes significantly to the morbidity and mortality of patients with cancer, with fatal PE being 3 times more common in patients with cancer than in those without cancer. Delayed initiation of anticoagulant treatment in PE correlates with increased mortality rates.^[3,4]^ The Pulmonary Embolism Severity Index is used to predict 30-day mortality in patients with PE, particularly those at low risk.^[5]^ The European Society of Cardiology classifies the initial severity of PE in patients based on hemodynamic stability, Pulmonary Embolism Severity Index score, right ventricular dysfunction, and elevated troponin levels, guiding treatment strategies such as thrombolysis or anticoagulation.^[6]^ Anticoagulant therapy increases the risk of bleeding, particularly in patients with cancer, with metastatic disease or gastrointestinal primaries conferring an even higher risk.^[7–9]^ Currently available bleeding risk prediction models, such as OBRI, Kuijer, RIETE, HAS-BLED, and VTE-BLEED, still show insufficient discriminative capacity to guide clinical decisions reliably.^[10–15]^ Therefore, we had to identify the optimal therapeutic strategy by carefully balancing the risks of thrombosis and bleeding.

According to the European Society of Cardiology guidelines, the patient was initially classified as having an intermediate-to-high risk PE, warranting full-dose anticoagulation due to the acuity, high clot burden, and active cancer. Due to the patient’s gastric cancer and associated high bleeding risk, non-vitamin K antagonist oral anticoagulants were not recommended, making low-molecular-weight heparin (LMWH) the optimal initial choice.^[16,17]^ LMWH has the advantage of flexible dosage adjustment for patients with thrombocytopenia, allowing for modifications based on the degree of thrombocytopenia. The International Society on Thrombosis and Haemostasis recommends a full therapeutic dose of anticoagulation for patients with cancer-associated venous thrombosis and PLT > 50 × 10^9^/L. For lower-risk patients, a consideration of using 50% or prophylactic dose of LMWH for PLT between 25 × 10^9^/L and 50 × 10^9^/L could be considered.^[17]^ However, the relative safety and efficacy of these approaches remain to be confirmed. Currently, 2 prospective studies have addressed anticoagulant treatment in patients with cancer and thrombocytopenia. The TROVE advocates for dose adjustment of anticoagulation in patients with cancer with reduced PLT, while the CAVEaT supports full-dose anticoagulation.^[18,19]^ Considering the patient’s heightened bleeding risk and low HB levels, we opted for a half-dose enoxaparin regimen for anticoagulation. Unfortunately, following the administration of half-dose anticoagulation, the patient experienced an escalation in gastrointestinal bleeding accompanied by a progressive decline in PLT, which significantly complicated anticoagulation management.

Based on the declining PLT trend following enoxaparin initiation, stable white blood cell count, and a 4T’s score of 4, HIT was suspected in this patient. Considering the potential risk of HIT, we decided to discontinue enoxaparin and switch to fondaparinux for anticoagulation therapy.^[20,21]^ The fondaparinux sodium dose was carefully titrated from 2 mg to 1 mg and then back to 2 mg daily based on gastrointestinal bleeding activity and HB levels, resulting in the resolution of both thrombocytopenia and PE.

Although we successfully treated a case of PE in a patient with gastric cancer complicated by gastrointestinal bleeding and HIT, certain limitations must be acknowledged. The patient presented with suspected HIT; however, owing to the lack of relevant laboratory testing resources at our medical institution, we were unable to confirm this through laboratory verification. Under the current circumstances, we strive to make the most precise clinical judgment by integrating the available information.

## 4. Conclusion

We present a case of gastric cancer in which gastrointestinal bleeding and HIT occurred during PE treatment. In this unique case, we successfully balanced the risk of bleeding and anticoagulation therapy by identifying and effectively addressing HIT. The treatment strategy involved transitioning from enoxaparin to fondaparinux and implementing a vena cava filter placement. By dynamically adjusting anticoagulation therapy based on the patient’s evolving condition and bleeding status at different stages, we successfully treated the patient’s PE, ensuring control of the risk of gastrointestinal bleeding and restoration of the PLT. Navigating the complexities of anticoagulation therapy, particularly in patients with advanced cancer with simultaneous bleeding and thrombocytopenia, requires individualized adjustment of anticoagulation treatment.

## Author contributions

**Funding acquisition:** Xiaolin Yun, Difei Yao.

**Project administration:** Caixia Dong.

**Data curation:** Wenjun Yang.

**Formal analysis:** Xiaozhao Zhuang.

**Validation:** Chenxin Zeng.

**Supervision:** Shuzhen Huang.

**Writing – review & editing:** Difei Yao.

**Writing – original draft:** Xiaolin Yun, Tiantian Wang.
